# Infants born large-for-gestational-age display slower growth in early infancy, but no epigenetic changes at birth

**DOI:** 10.1038/srep14540

**Published:** 2015-09-30

**Authors:** Valentina Chiavaroli, Wayne S. Cutfield, José G. B. Derraik, Zengxiang Pan, Sherry Ngo, Allan Sheppard, Susan Craigie, Peter Stone, Lynn Sadler, Fredrik Ahlsson

**Affiliations:** 1 Liggins Institute, University of Auckland, Auckland, New Zealand; 2 Department of Obstetrics and Gynaecology, Faculty of Medical and Health Sciences, University of Auckland, Auckland, New Zealand; 3 National Women’s Health, Auckland District Health Board, Auckland, New Zealand

## Abstract

We evaluated the growth patterns of infants born large-for-gestational-age (LGA) from birth to age 1 year compared to those born appropriate-for-gestational-age (AGA). In addition, we investigated possible epigenetic changes associated with being born LGA. Seventy-one newborns were classified by birth weight as AGA (10^th^–90^th^ percentile; n = 42) or LGA (>90^th^ percentile; n = 29). Post-natal follow-up until age 1 year was performed with clinical assessments at 3, 6, and 12 months. Genome-wide DNA methylation was analysed on umbilical tissue in 19 AGA and 27 LGA infants. At birth, LGA infants had greater weight (p < 0.0001), length (p < 0.0001), ponderal index (p = 0.020), as well as greater head (p < 0.0001), chest (p = 0.044), and abdominal (p = 0.007) circumferences than AGA newborns. LGA infants were still larger at the age of 3 months, but by age 6 months there were no more differences between groups, due to higher length and weight increments in AGA infants between 0 and 6 months (p < 0.0001 and p = 0.002, respectively). Genome-wide analysis showed no epigenetic differences between LGA and AGA infants. Overall, LGA infants had slower growth in early infancy, being anthropometrically similar to AGA infants by 6 months of age. In addition, differences between AGA and LGA newborns were not associated with epigenetic changes.

There is no universal definition of oversize at birth. Nonetheless, babies born large-for-gestational-age (LGA) are usually defined by weight, determined as >90^th^ percentile at birth according to gestational age and sex, although the 95^th^ or 97^th^ percentile have also been used. A systematic review and meta-analysis showed that high birth weight is independently associated with increased overweight risk in childhood and adulthood^1^. In addition, epidemiological studies have shown a strong association between being born LGA and later adverse metabolic outcomes, including type 2 diabetes, and other cardiovascular disorders^2^,^3^,^4^. The underlying mechanisms and developmental pathways to later disorders are still unclear, but both intrauterine environmental factors^5^ and early postnatal events^6^ seem to be involved.

More than three decades ago, Davies *et al.* reported rapid downwards shift in length increase during the first 3 months of life in LGA infants, as well as a slower than average weight gain in the first 6 months^7^. More recently, Taal *et al.* confirmed a ‘catch-down’ growth in both weight and length in LGA infants, mainly occurring during the first 3 months of life, leading to a substantial realignment on all growth parameters compared to infants born appropriate-for-gestational-age (AGA)^8^. Thus, it has been speculated that, after escaping the strong maternal influence on intrauterine growth, LGA infants return to their genetically-determined growth trajectory^7^. In contrast however, other studies showed that infants born LGA were 4.6 and 2.2 times more likely to be overweight at 6 and 12 months of age than AGA infants^9^. In addition, greater central adiposity has been found at age 12 months in those LGA infants born of mothers with gestational diabetes^10^. Therefore, based on the wide heterogeneity of the available evidence, it is difficult to reach firm conclusions on the postnatal growth trajectories in infants born LGA^7^,^8^,^9^,^10^.

Sustained changes in growth and metabolism following an adverse fetal or early neonatal environment have been associated with mechanisms involving environmental regulation of gene expression^11^. Environmental factors can trigger long-lasting changes through these epigenetic processes, which regulate gene expression without affecting the genetic sequence^12^,^13^,^14^, such as DNA methylation^15^. There is a large number of animal studies showing that manipulation of the early life environment is associated with the development of adverse cardio-metabolic outcomes later in life^16^,^17^. The possible link between epigenetic regulation in fetal tissues and intrauterine growth restriction has also been investigated^18^,^19^. Notably, specific epigenetic changes have been linked to growth restriction, including alterations in genomic imprinting and DNA methylation^20^. However, the potential association between DNA methylation and high birth weight has not been adequately explored, and only very recently a candidate gene (*FGFR2*) has been identified^21^.

Thus, in the present study we aimed to evaluate the growth patterns of infants born LGA from birth to age 1 year in comparison to those born AGA. In addition, we aimed to assess whether there were epigenetic changes at birth associated with being born LGA.

## Patients and Methods

### Ethics approval

Ethics approval for this study was provided by the Northern Y Regional Ethics Committee (Ministry of Health, New Zealand). Written informed consent was obtained from parents or guardians. This study was performed in accordance with all appropriate institutional and international guidelines and regulations for medical research, in line with the principles of the Declaration of Helsinki.

### Participants

This study involved a prospective cohort of healthy infants recruited at birth from the Newborn Services, Auckland City Hospital (New Zealand), between March and September 2011. All infants were born at term (37–41 weeks of gestation) from singleton and uneventful pregnancies. Infants were excluded if conceived by *in vitro* fertilisation, or born to mothers with type 1 diabetes or gestational diabetes, preeclampsia, gestational or pre-existing hypertension, chronic illnesses, or following maternal use of recreational drugs, tobacco, or alcohol during pregnancy. Other exclusion criteria were chromosomal or single gene defects, syndromal diagnosis, as well as having a first-degree relative or grandparent with diabetes or the metabolic syndrome.

### Neonatal clinical assessment

All neonatal auxological measures were obtained by a single study investigator within 48 h of birth. These included weight, crown-heel length, as well as head, chest and abdominal circumferences. Birth weight was measured to the nearest 10 g using electronic infant scales, and birth weight data were transformed into standard deviations scores (SDS)^22^. Crown-heel length was measured using a neonatometer (Holtain Ltd., Crymych, U.K.), and circumferential measurements were obtained to the nearest millimetre. Body mass index (BMI) and ponderal index were calculated as markers of adiposity. The study population was divided into two groups according to birth weight: infants born AGA (birth weight between the 10^th^ and 90^th^ percentiles) or LGA (birth weight greater than the 90^th^ percentile)^23^.

Gestational age was determined by hierarchical integration of the following variables: date of last menstrual period, menstrual cycle length, ultrasound primarily at 16–20 weeks, and clinical assessment of gestational age at birth^24^,^25^. BMI at birth was calculated as per standard formula (kg/m^2^), with SDS corrected for sex only^26^. BMI SDS was derived according to British 1990 standards^27^. Ponderal index was calculated as birth weight in grams (×100) divided by the cube of the crown-heel length in centimetres^28^.

Maternal obstetric history was recorded to clarify age at time of delivery, mode of delivery (vaginal delivery or caesarean section), parity, and the birth order of each infant. Maternal and paternal weights and heights, maternal pre-pregnancy weight and BMI, and weight and BMI at the end of pregnancy were obtained. Mean parental BMI was calculated as the average of maternal and paternal BMI. Mid-parental height SDS was calculated using standard formulae^29^. Ethnicity was recorded by self-report using a prioritized system, such that if multiple ethnicities were selected, the patient was assigned to a single category, following a hierarchical system of classification^30^.

Umbilical cord tissue was collected at birth, and frozen at −80 °C for later genome-wide DNA methylation analysis.

### Longitudinal clinical assessment

All participants were re-evaluated by the same study investigator at 3, 6, and 12 months of age. Anthropometric measures, including weight, length, as well as head, chest, and abdominal circumferences were measured.

### Genome-wide methylation assay

Genomic DNA was isolated from umbilical tissue samples using the KingFisher Cell and Tissue DNA kit (Thermo Fisher Scientific, Vantaa, Finland) according to manufacturer’s instructions. One μg of DNA was bisulfite treated using the EZ DNA Methylation™ Kit (Zymo Research, Orange, CA, USA), and 500 ng of bisulfite-treated DNA was analysed using the Illumina Infinium 450 K methylation array platforms. To assess DNA methylation profile, we used the standard Illumina protocols. The bisulfide-converted samples were whole-genome amplified, and the amplified products were fragmented by an endpoint enzymatic process. The fragmented DNA was purified and hybridized to the Infinium Human Methylation 450 K BeadChips (Illumina Inc., San Diego, CA, USA). During hybridization, the amplified and fragmented DNA samples anneal to locus-specific DNA oligomers residing on the bead chips. Single base extension reaction, washing, and staining were carried out using a TECAN Te-Flow chamber. The stained arrays were assessed for fluorescence intensities at the methylated and unmethylated bead sites using Illumina BeadArray Reader (Illumina Inc.). Quality control was performed using built-in controls. All samples passed quality control criteria (<5% of probes were invalid). DNA methylation signals (β-values) from the scanned arrays were extracted using the methylation module of GenomeStudio® software version 2011.1 (Illumina Inc.). The β-value was used to estimate the methylation level of the CpG locus by calculating the ratio of intensities between methylated and unmethylated alleles (1 > average β > 0 represents fully methylated to un-methylated alleles). R package Lumi^31^ and wateRmelon^32^ were used for sample quality filtering and assessment, for all β-values pre-processing, correction and normalisation steps. Differential methylation analysis to determine an association between CpG methylation at each site as a function of the phenotype of interest (LGA versus AGA) was performed using the R package CpGassoc^33^ and RnBeads^34^.

### Statistical analyses

Demographic characteristics between groups were compared with one-way ANOVA and Fisher’s exact tests. Anthropometric differences between groups were analysed using general linear regression models. Models accounted for important confounding factors (gestational age, sex, ethnicity). Models examining differences between LGA and AGA infants at 3, 6, and 12 months included age as a covariate. Binary logistic regressions were carried out to examine the factors affecting the likelihood of being born LGA or undergoing a caesarean section. Statistical analyses were carried out in SAS version 9.3 (SAS Institute Inc. Cary NC, USA) and Minitab v.16 (Pennsylvania State University, State College, PA, USA). All statistical tests were two-tailed and maintained at a 5% significance level. Demographic data are presented as means ± standard deviations. Outcome data are presented as model-adjusted means (estimated marginal means adjusted for the confounding factors in the models), with associated 95% confidence intervals.

For statistical analysis on differential methylation levels, probes with detection p-value > 0.05 were excluded. CpG sites with missing data for >10% of samples were also excluded from analysis. All CpGs residing on X and Y chromosomes were dropped from the analysis to eliminate systematic sex differences. We performed a multivariate linear mixed regression analysis that modelled the β-values as the dependent variables, with the variable group (AGA or LGA) as the primary independent variable, and including covariates for sex, birth weight, and ethnicity to adjust for potential effects of other covariate-dependent methylation. To assess significance while accounting for multiple testing, we used the Benjamini-Hochberg false discovery rate (FDR) procedure^35^. Statistical significance of the differentially methylated CpGs was determined using the FDR with a cut-off of 0.05 to correct for multiple hypothesis testing. Running parallel to the CpGassoc method, a linear modelling using the limma^36^ package in RnBeads was used for computing the CpGs site specific p-values, with a FDR cut-off of 0.05.

## Results

### Study cohort

Eligible pregnant women in early labour were recruited from the Delivery Suite at Auckland City Hospital. We aimed to recruit at a ratio of approximately two controls for each LGA participant. Of 57 LGA and 108 AGA babies eligible at birth, seventy-one infants (43%) attended all follow-up assessments (at 3, 6, and 12 months of age) and were included in this study: 42 born AGA (18 boys) and 29 born LGA (18 boys) (Supplementary Figure 1). The primary reasons for losing infants from the original cohort to the longitudinal part of the study were living outside the Auckland region, being uncontactable, and lack of parental interest in ongoing participation in the study. However, AGA participants were similar in all birth and parental characteristics to AGA infants excluded (data not shown). The same applied to LGA subjects, except that LGA participants had greater abdominal circumference than LGA infants excluded (p = 0.004).

The parents of LGA children were considerably fatter than the parents of AGA infants, with a 2.4 kg/m^2^ difference in mean parental BMI (p = 0.004). Prior to pregnancy, LGA mothers had BMI that was 2.7 kg/m^2^ greater than mothers of the AGA group (p = 0.004). Not surprisingly, every 1 kg/m^2^ increase in maternal pre-pregnancy BMI was associated with an 11% increase in the odds of a LGA infant being born (p = 0.014). In addition, increasing birth order was associated with an increased likelihood of LGA birth (odds ratio 1.66; p = 0.034) (Table 1). The rate of delivery by caesarian section was 21% among those born LGA in comparison to 10% for the AGA group (odds ratio 3.0; p = 0.036).

### Auxology at birth

At birth, LGA were larger than AGA infants based on all parameters assessed (Table 2). LGA infants were 800 g heavier (p < 0.0001), 2.7 cm longer (p < 0.0001), with a ponderal index 0.15 g*100/cm^3^ greater (p = 0.020) than AGA infants. Further, LGA infants had greater circumference of the head (+1.9 cm; p < 0.0001), chest (+0.9 cm; p = 0.044), and abdomen (+1.5 cm; p = 0.007).

### Longitudinal assessment over the first year of life

By 3 months of age, there were persisting differences between LGA and AGA infants. LGA subjects were still 1.5 cm longer (p = 0.006), 713 g heavier (p < 0.0001), and of BMI 1 kg/m^2^ greater (p = 0.030), and had greater head (+1.1 cm; p = 0.004) and abdominal (+1.4 cm; p = 0.042) circumferences. However, there were no significant differences in ponderal index or chest circumference.

**Figure 1:**
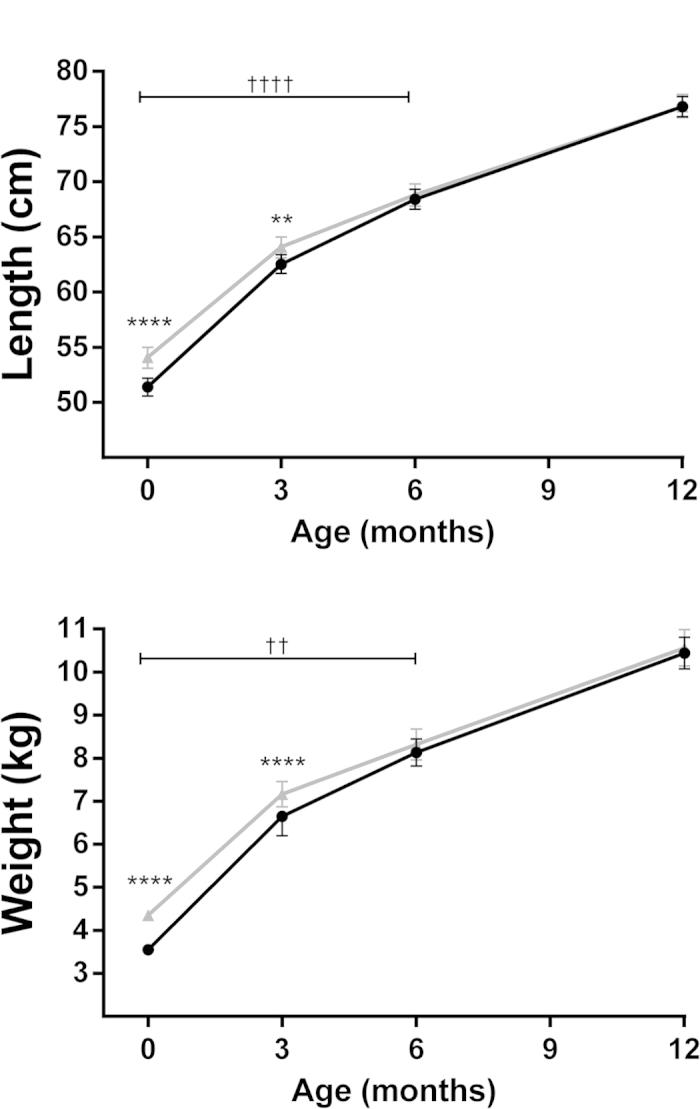
Length and weight increments in AGA (continuous) and LGA (dashed) infants from birth to 12 months of age. Data are means and 95% confidence intervals adjusted for confounding factors in the multivariate models. **p < 0.01 and ****p < 0.0001 for differences at a given time point; ^††^p < 0.01 and ^††††^p < 0.0001 for differences in length or weight velocity.

In the first 6 months of life, LGA infants grew significantly slower than those born AGA: length increment 15.1 vs 18.3 cm, respectively (p < 0.0001) and weight increment 4.13 vs 4.87 kg, respectively (p = 0.002) (Fig. 1). These differences accounted for the fact that both groups were anthropometrically similar by 6 months of age. LGA and AGA infants were still similar at the one-year follow-up (Table 3), as length and weight increments were virtually identical in the two groups between 6 and 12 months of age (7.7 cm and 2.1 kg for both groups).

### Genome-wide methylation analysis

**Figure 2:**
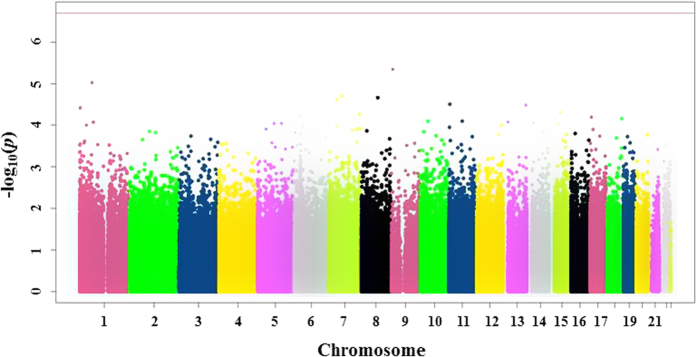
Graphic output of the CpGassoc method, showing the Manhattan plot for the association between methylation and AGA-LGA group. X-axis: location of CpG site in the genome by chromosome; y-axis: -log10 of the p-value for each CpG site (dots), with more negative values indicating greater differences between groups. The red horizontal line at the top of the figure represents the cutoff for FDR-adjusted p < 0.05; the absence of dots above this line shows that no statistically significant differences were observed.

The genome-wide methylation analysis was carried out on samples from 46 infants, including 19 AGA and 27 LGA infants. For this analysis, more than 485,000 DNA methylation sites covering 99% of human NCBI Reference Sequence (RefSeq) genes were examined at birth. 449,691 probes (92.6%) out of 485,577 passed the probe filtering criteria. The differential methylation analysis of CpGs sites showed no significant differences between LGA and AGA infants at birth (all p > 0.05) (Fig. 2 and Supplementary Figure 2).

## Discussion

Our results indicate that, despite being born oversized, LGA infants displayed slower length and weight velocity, so that by the age of 6 months LGA infants were anthropometrically similar to AGA infants. This suggests that LGA infants experience a slowing in growth in early infancy. In addition, no epigenetic differences in genome-wide methylation were found in LGA infants at birth.

Alterations in the intrauterine environment can induce fetal developmental adaptations that might have long-lasting detrimental effects on the offspring^37^. Maternal factors exert a critical role in determining the overall health of newborns, with maternal obesity and gestational diabetes accounting for a marked increase in the number of LGA infants^5^,^38^. Nonetheless, a considerable number of these infants are born to healthy and normoglycemic women^38^, and in such cases the underlying mechanisms for oversize at birth are yet to be identified.

As mentioned previously, there is a paucity of data on growth outcomes of LGA infants. Our data corroborate previous studies showing that LGA newborns generally present greater adiposity besides being born oversized^39^,^40^. We observed that LGA subjects had greater ponderal index and abdominal circumference than AGA infants at at birth, and still greater abdominal circumference at 3 months of age. Although both are indirect measures of neonatal adiposity, they provide useful information on newborn body fat mass^41^.

Nevertheless, we observed a subsequent slower growth in LGA infants, culminating in similar anthropometry in LGA and AGA groups by 6 months of age, which remained so at 12 months. These findings are in line with previous data showing a slowdown in weight and length gain from birth to age 6 months in most LGA infants^7^,^8^. In order to explain the reduced growth *ex utero* in those born LGA, a putative role has been attributed to maternal influences to account for their increased growth *in utero*. For instance, fetal overgrowth in oversized infants of non-diabetic mothers has been linked to an energy-rich fetal environment associated with mild maternal hyperglycemia (below the cut-off levels for gestational diabetes), maternal obesity, or excessive gestational weight gain^42^,^43^. Thus, it is speculated that after birth LGA infants are free from these intrauterine stimuli, leading to the natural expression of their genetic growth patterns.

However, the data on LGA infants are inconsistent and our results are in contrast with some studies reporting greater central adiposity at age 12 months in those born LGA compared to AGA infants^9^,^44^. In particular, Vohr *et al.* reported a unique pattern of adiposity (based on a higher BMI, abdominal circumference and abdominal skinfold) at birth and still at age 12 months in LGA infants of diabetic mothers, in comparison to LGA of healthy mothers and AGA infants of diabetic mothers, supporting the additional detrimental effects of maternal diabetes on offspring growth^10^. As a result, we excluded infants born to mothers with gestational diabetes or who had a first-degree relative or grandparent with diabetes, in order to minimize the potential effects of such confounders. This may explain the lack of observed differences between AGA and LGA from 6 months of age in our study.

There is a large body of evidence showing that changes in the early life environment are associated with increased cardio-metabolic risk later in life^16^,^17^,^45^. Epigenetics (a dynamic process that alters gene expression without changes in DNA sequence) has been linked to the regulation of fetal tissue growth and development^18^,^19^, and epigenetic changes have been associated with aberrant intrauterine growth^20^,^46^. Genes such as *IGF2* and *H19* are known to affect the fetal development^47^, and epigenetic modifications in their imprinting control regions can therefore affect fetal growth. For example, hypomethylation has been associated with a repressed *IGF2* expression responsible for pre- and post-natal growth restriction (roughly 30% of Silver Russell syndrome cases), while hypermethylation induces *IGF2* overexpression leading to overgrowth (Beckwith-Wiedemann syndrome)^47^. There is also increasing evidence that a range of genes associated with an increased risk of obesity are susceptible to epigenetic changes^48^.

Potential epigenetic changes *in utero* associated with the LGA phenotype, which could possibly lead to heavier birth weight and altered body composition and metabolism, have only recently been examined^21^. Methylation at three CpGs in the *FGFR2* gene were identified as being associated with high birth weight^21^. However, we observed no epigenetic alterations in genome-wide methylation analysis at birth in LGA infants. It has to be acknowledged that we undertook the DNA methylation analysis in cord samples from a subgroup of AGA and LGA newborns, and it is conceivable that there is differential DNA methylation in other tissues. However, it is not feasible to sample DNA from multiple organs and tissues in newborn infants. In addition, cord samples have been found to be advantageous as they provide a great amount of fetal mesenchymal cells and vascular tissue^49^. Nonetheless, further studies are needed to assess whether umbilical cord tissues can indeed be used as reliable markers of DNA methylation in other tissues.

In conclusion, our study showed that although LGA babies were larger and had greater adiposity at birth, a slowing in growth (length and weight) occurs in these infants in early infancy, leading to a similar anthropometry to AGA infants by 6 months of age. Of note, the differences observed in early life between LGA and AGA infants were not associated with epigenetic changes. Further research is needed to clarify the growth pattern of those born LGA in early infancy and childhood, and to elucidate possible mechanisms associated with overgrowth *in utero*.

## Additional Information

**How to cite this article**: Chiavaroli, V. *et al.* Infants born large-for-gestational-age display slower growth in early infancy, but no epigenetic changes at birth. *Sci. Rep.* **5**, 14540; doi: 10.1038/srep14540 (2015).

## Supplementary Material

Supplementary Information

## Figures and Tables

**Table 1:** Demography of the study population and parental features.

	AGA	LGA	p-value
n	42	29	
Demography			
Sex ratio (boys:girls)	18:24	18:11	0.15
Ethnicity (New Zealand European)	57%	55%	0.99
Gestational age (weeks)	40.1 ± 1.2	40.1 ± 0.9	0.83
Birth order (first-borns)	45%	28%	0.15
Parental characteristics			
Maternal age at childbirth (years)	33.1 ± 4.2	31.7 ± 5.7	0.24
Maternal pre-pregnancy BMI (kg/m^2^)	23.4 ± 4.4	26.1 ± 4.9	**0.004**
Mean parental BMI (kg/m^2^)	24.9 ± 3.0	27.3 ± 4.5	**0.004**

Gestational age and parental data are means ± standard deviations.

**Table 2:** Characteristics of the study population at birth.

	AGA	LGA	p-value
n	42	29	
Anthropometry			
Birth weight (kg)	3.55 (3.44–3.65)	4.35 (4.23–4.47)	**<0.0001**
Birth weight SDS	0.23 (−0.01–0.47)	1.92 (1.65–2.19)	**<0.0001**
Birth length (cm)	51.4 (50.6–52.2)	54.1 (53.1–55.0)	**<0.0001**
Ponderal index (g/cm^3^ × 100)	2.61 (2.52–2.71)	2.76 (2.65–2.86)	**0.020**
BMI (kg/m^2^)	13.4 (13.1–13.7)	14.9 (14.5–15.3)	**<0.0001**
Head circumference (cm)	35.0 (34.5–35.4)	36.9 (36.4–37.4)	**<0.0001**
Chest circumference (cm)	36.2 (35.5–37.0)	37.1 (36.4–37.9)	**0.044**
Abdominal circumference (cm)	35.5 (34.7–36.4)	37.0 (36.2–37.9)	**0.007**

Data are means and 95% confidence intervals adjusted for other confounding factors in the multivariate models.

**Table 3:** Characteristics of the study population at the one-year follow-up.

	AGA	LGA	p-value
n	42	29	
Age (days)	373 ± 17	367 ± 12	0.10
Anthropometry			
Weight (kg)	10.44 (10.08–10.81)	10.57 (10.14–10.99)	0.61
Length (cm)	76.8 (75.9–77.7)	76.8 (75.8–77.9)	0.90
Length SDS	0.75 (0.38–1.12)	0.78 (0.35–1.21)	0.90
Ponderal index (g/cm^3^ × 100)	2.31 (2.22–2.39)	2.33 (2.23–2.43)	0.71
Body mass index (kg/m^2^)	17.7 (17.1–18.2)	17.9 (17.2–18.5)	0.58
Chest circumference (cm)	48.4 (46.2–50.7)	46.6 (44.0–49.2)	0.23
Abdominal circumference (cm)	46.7 (45.4–48.0)	47.0 (45.5–48.5)	0.72

Age data are means ± standard deviation; other data are means and 95% confidence intervals adjusted for other confounding factors in the multivariate models.
