# Supplementary material for: Infants born large-for-gestational-age display slower growth in early infancy, but no epigenetic changes at birth
Source: Sci Rep. 2015 Sep 30;5:14540. doi: 10.1038/srep14540 (PMC4588582; doi:10.1038/srep14540)

Infants born large-for-gestational-age display slower growth in early infancy, but no epigenetic changes at birth

Valentina Chiavaroli[1,*], Wayne S Cutfield[1], José G B Derraik[1], Pan Zengxiang[1], Sherry Ngo[1], Allan Sheppard[1], Susan Craigie[1], Peter Stone[3], Lynn Sadler[4], Fredrik Ahlsson[1,2]

Supplementary Figure Legends

Supplementary Figure 1. Summary of study's recruitment, with number of participants assessed at each time point.

Supplementary Figure 2. Output for RnBeads method, showing scatter (left) and volcano (right) plots for different methylation sites in AGA and LGA infants. In the scatter plot, x-axis and y-axis represent the mean β-values for AGA and LGA infants, respectively. In the volcano plot, x-axis shows the absolute difference in methylation (expressed as a ratio) for each CpG site, while the y-axis shows the -log10 of the p-value for the methylation difference at each CpG site between AGA and LGA infants. Red color would indicate a statistically significant difference (adjusted $p<0.05$), but the absence of red points in both scatter and volcano plots shows that no statistically significant associations were observed.

Supplementary Figure 1.

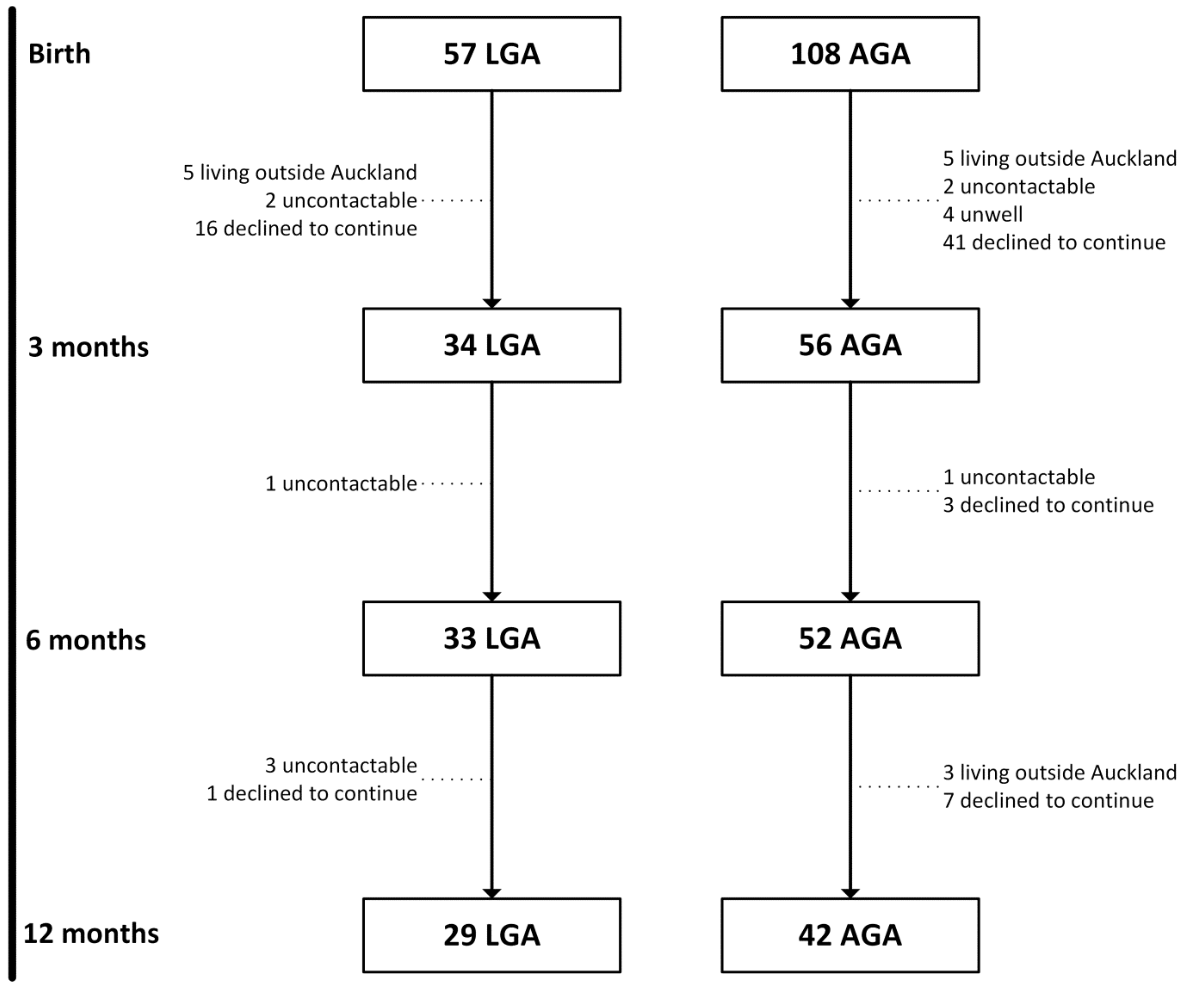

Supplementary Figure 2.

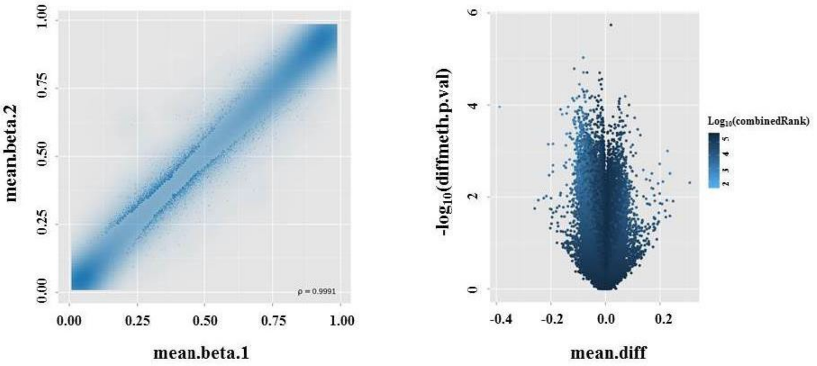

Supplement: Supplementary Information [file srep14540-s1.pdf]
